# Supplementary material for: Towards low-temperature processing of efficient γ-CsPbI3 perovskite solar cells
Source: J Mater Chem A Mater. 2023 Jul 10;11(30):16115–26. doi: 10.1039/d3ta03249c (PMC10394668; doi:10.1039/d3ta03249c)
Supplement: TA-011-D3TA03249C-s001 [file TA-011-D3TA03249C-s001.pdf]

Electronic Supplementary Material (ESI) for Journal of Materials Chemistry A.
This journal is © The Royal Society of Chemistry 2023

Supporting information

Towards low-temperature processing of efficient γ-$CsPbI_3$ perovskite solar cells

Zongbao Zhang,[a,b] Ran Ji, [a,b] Yvonne J. Hofstetter, [a,b] Marielle Deconinck,[a,b] Julius Brunner, [a,b] Yanxiu Li, [a,b] Qingzhi An, [a,b] Yana Vaynzof* [a,b]

[a] Chair for Emerging Electronic Technologies, Technische Universität Dresden, Nöthnitzer Straße 61, 01187 Dresden, Germany.
[b] Leibniz-Institute for Solid State and Materials Research Dresden, Helmholtzstraße 20, 01069 Dresden, Germany

E-mail: yana.vaynzof@tu-dresden.de

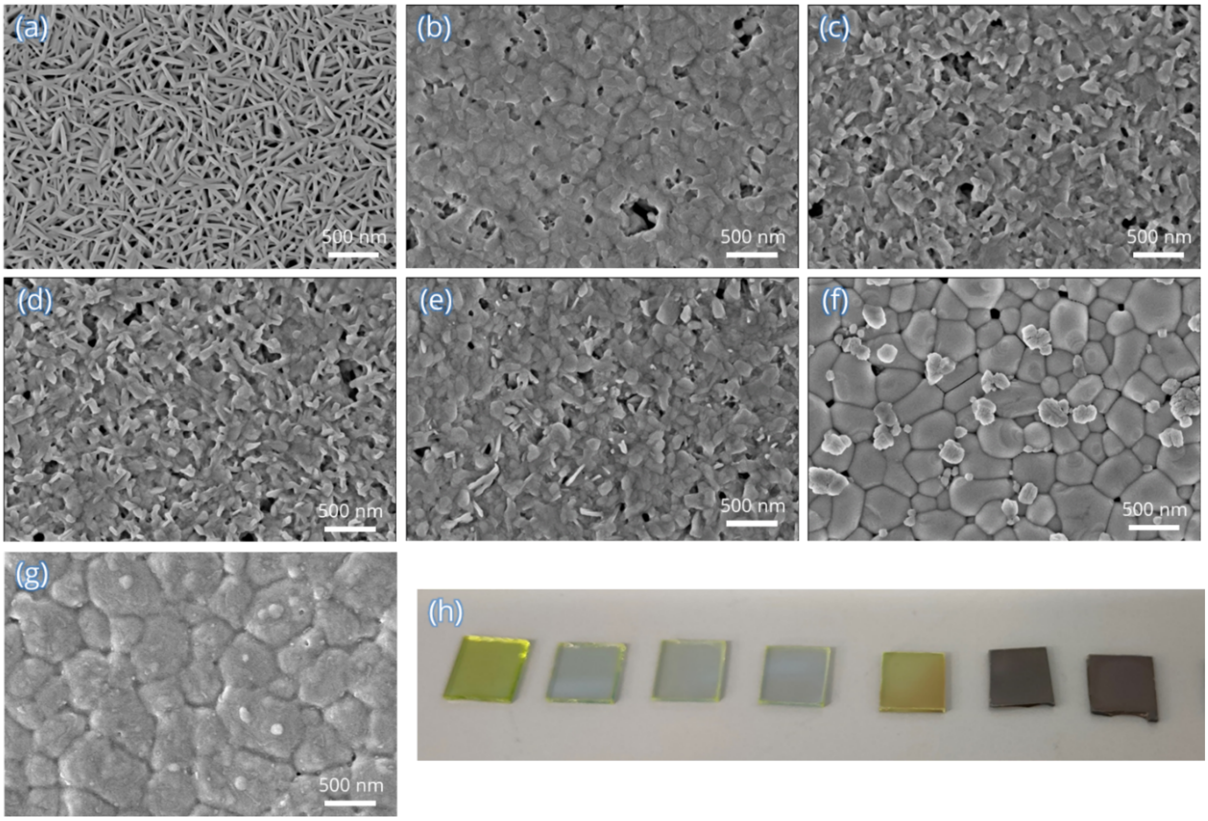

Fig. S1 Top-view SEM of perovskite with the aid of $Pb(OAc)_2$ and $EDAI_2$ undergoing different thermal annealing temperatures: (a) as-deposited wet film, (b) preheat at 60 ℃ for 1 min, (c) preheat at 60 ℃ for 3 min, (d) preheat at 60 ℃ for 5 min, (e) annealing at 180 ℃ for 5 s, (f) annealing at 180 ℃ for 30 s, (g) annealing at 180 ℃ for 3 min. And corresponding the film images are shown in (h).

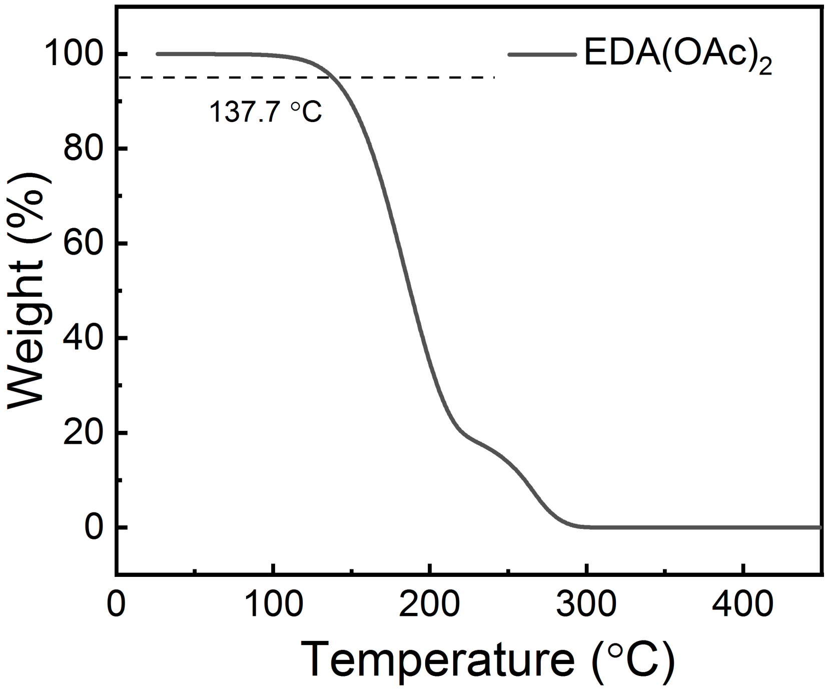

Fig. S2 TGA curves for $EDA(OAc)_2$ in nitrogen atmosphere.

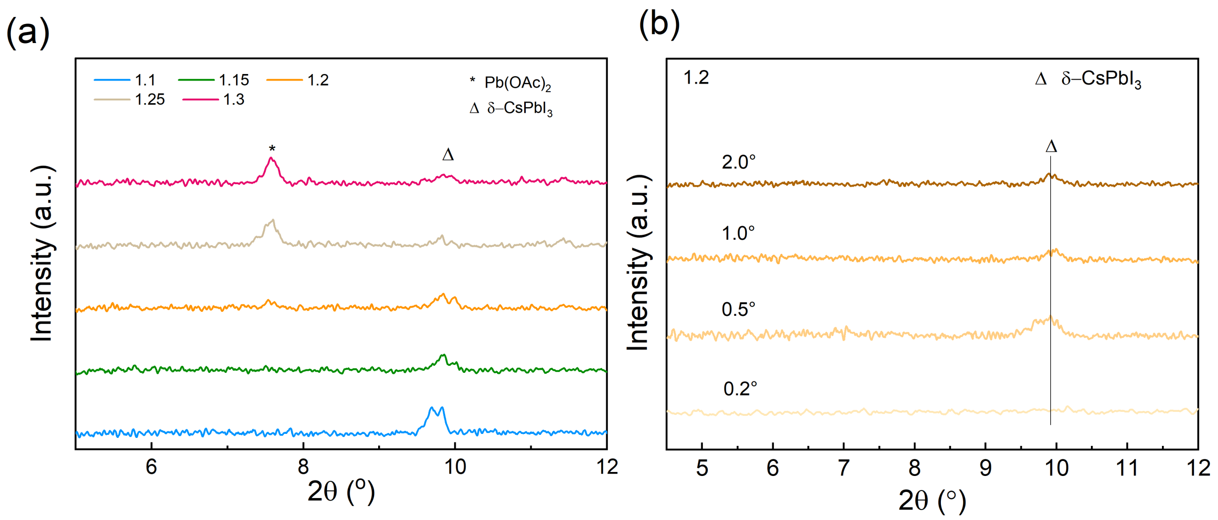

Fig. S3 (a) XRD patterns of $CsPbI_3$ with different $Pb(OAc)_2$ ratios at the low angle range, (b) Grazing incidence x-ray diffraction (GIXRD) patterns of 1.2 $CsPbI_3$ samples with different Ω values at low angles.

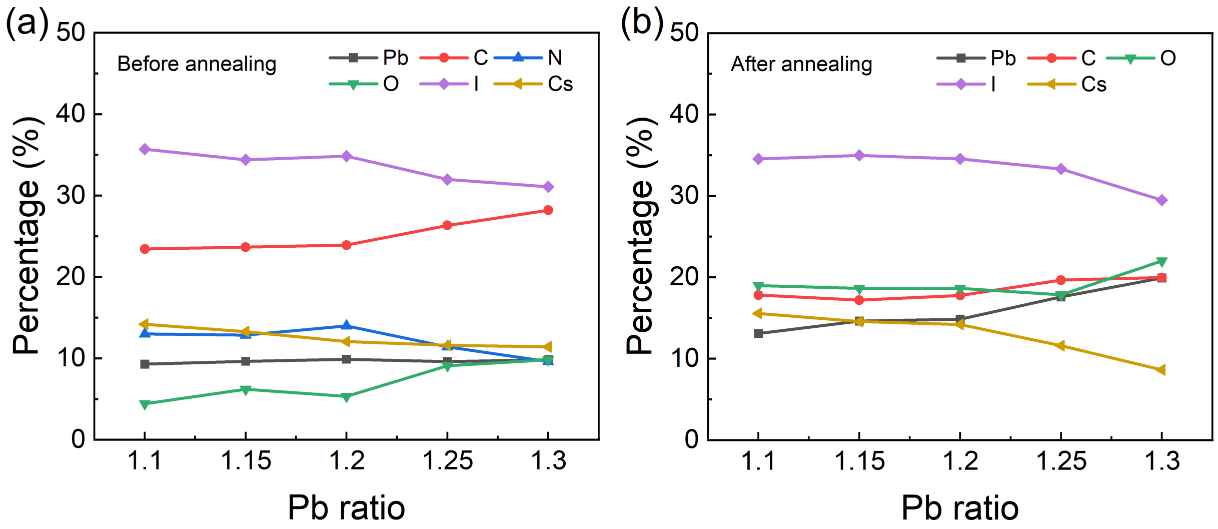

Fig. S4 Element concentration for different ratios of $Pb(OAc)_2$ (a) after preheating at 60 °C, but before annealing at 180 °C and (b) after annealing at 180 °C for 3 minutes.

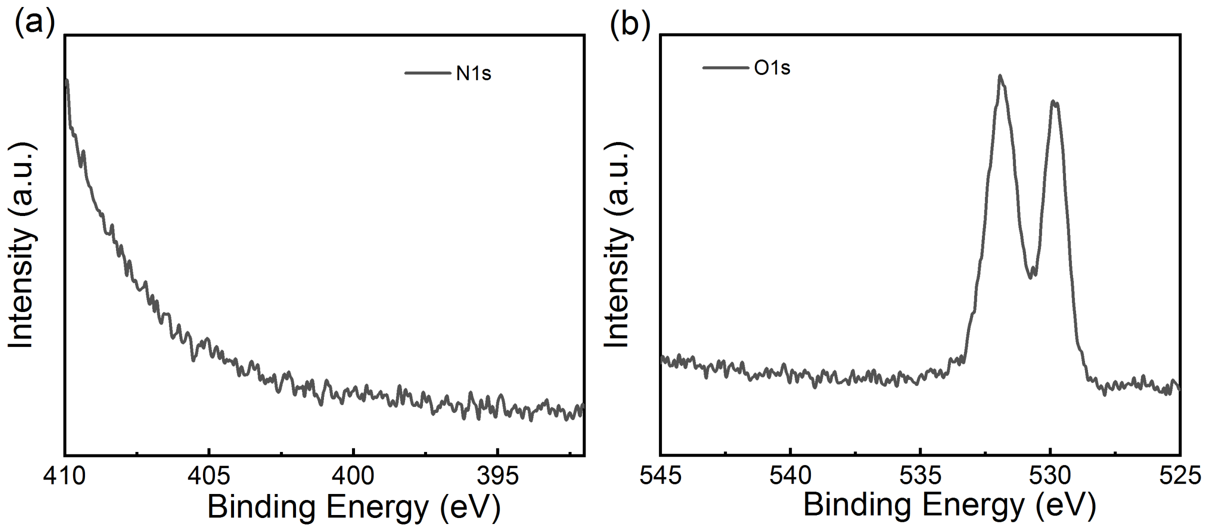

Fig. S5 (a) N1s and (b) O1s XPS spectra in 1.2 $CsPbI_3$ film after annealing.

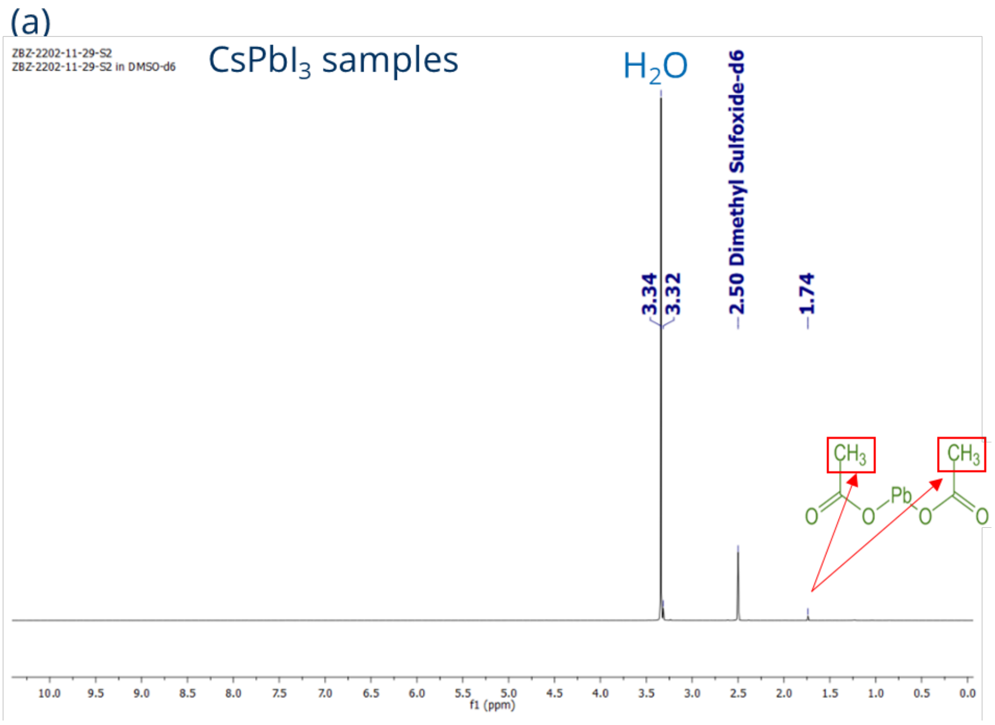

(b)

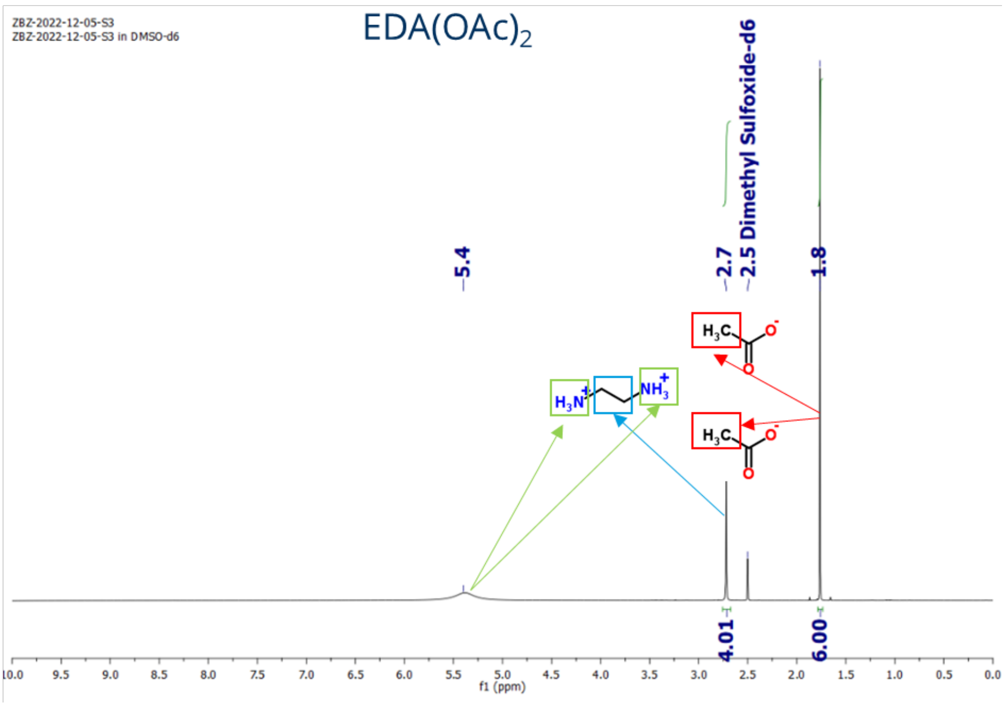

Fig. S6 H-NMR of final $CsPbI_3$ and intermediate $EDA(OAc)_2$ products in DMSO-D6 solvent. For the $CsPbI_3$ samples, scratched powder from the film dissolves in the DMSO-D6 solvent.

(a)

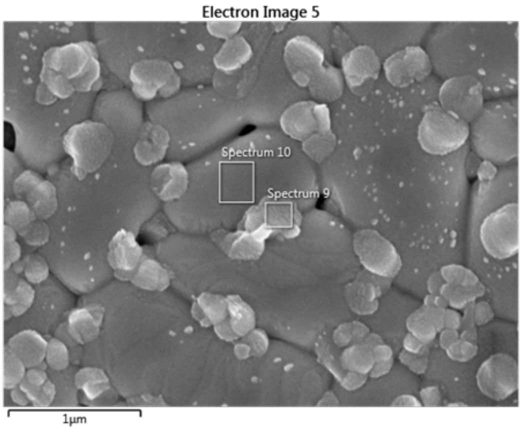

(b)

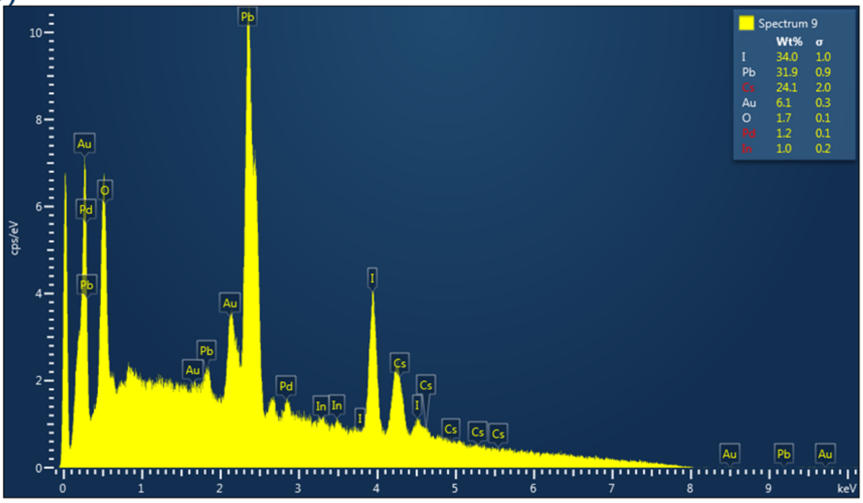

(c)

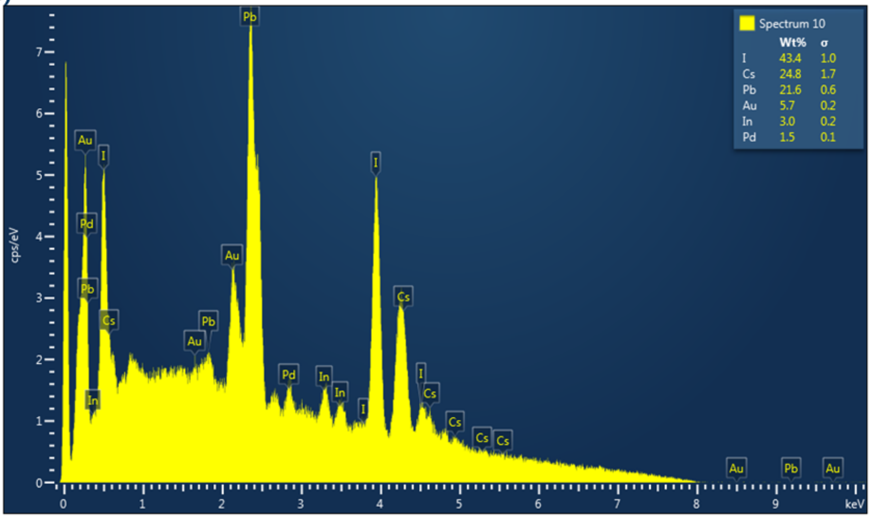

Fig. S7 (a) Top-view SEM images and (b), (c) corresponding EDX element composition spectra in 1.25 samples.

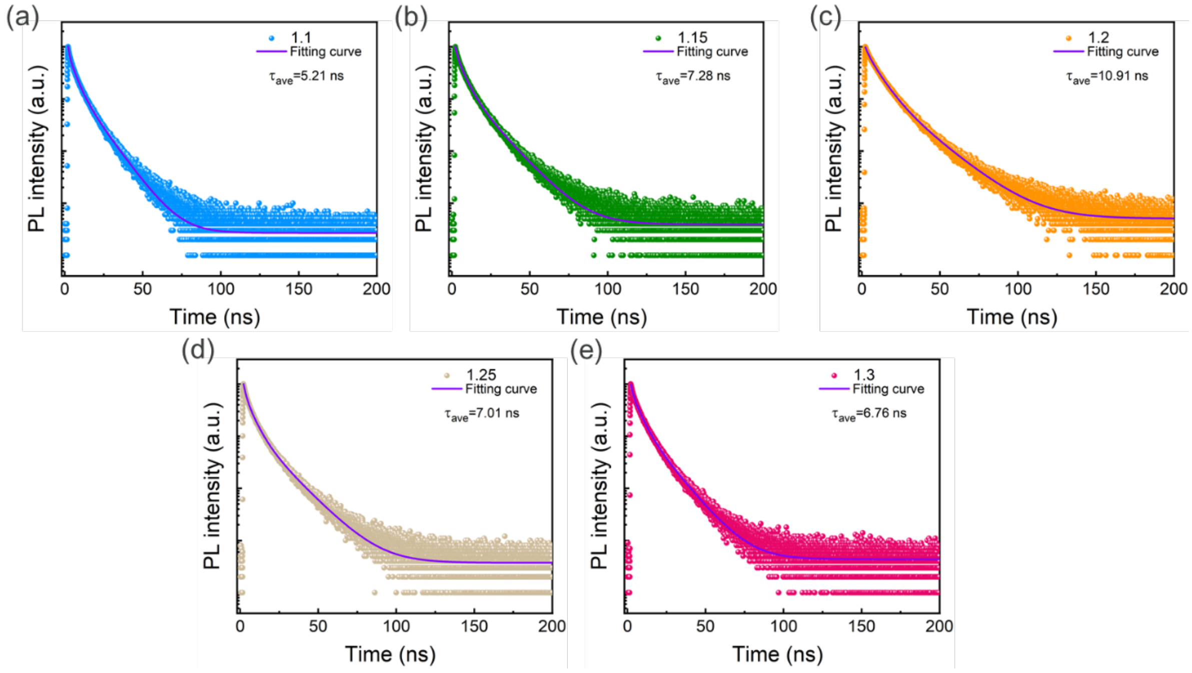

Fig. S8 TRPL fitting curves for different amount of $Pb(OAc)_2$: (a) 1.1, (b) 1.15, (c) 1.2, (d) 1.25, (e) 1.3. The carrier lifetime was obtained by fitting TRPL curves using the following formula: $y = A_1e^{-x_1/\tau_1} + A_2e^{-x_2/\tau_2} + A_3e^{-x_3/\tau_3} + B$. The average carrier lifetime was calculated by the equation: $\tau_{ave} = (\sum A_i\tau_i^2)(\sum A_i\tau_i)$. The related parameters are summarized in the Table S2.

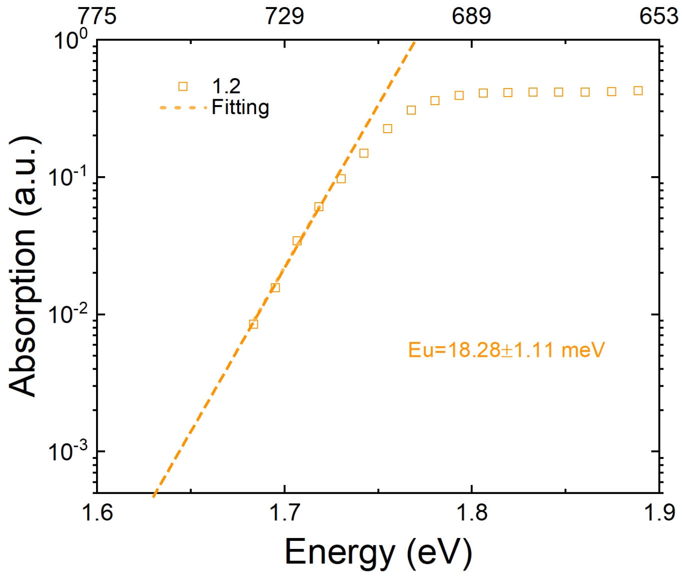

Fig. S9 Photothermal deflection spectroscopy (PDS) of 1.2 sample.

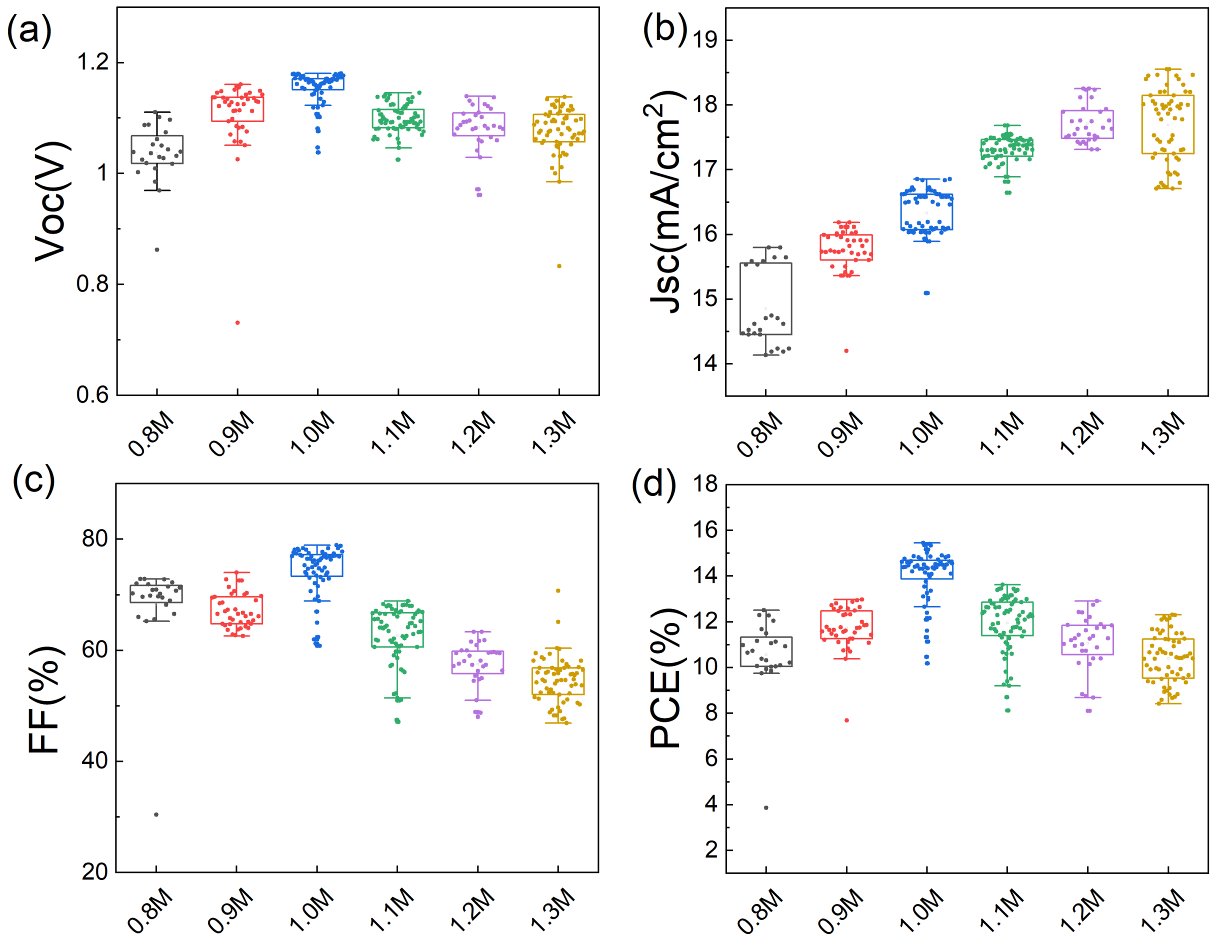

Fig. S10 Photovoltaic performance parameters: (a) V_{OC}, (b) J_{SC}, (c) FF and (d) PCE distribution of different perovskite precursor concentrations in 1.2 $CsPbI_3$ devices. A total of 290 devices were measured.

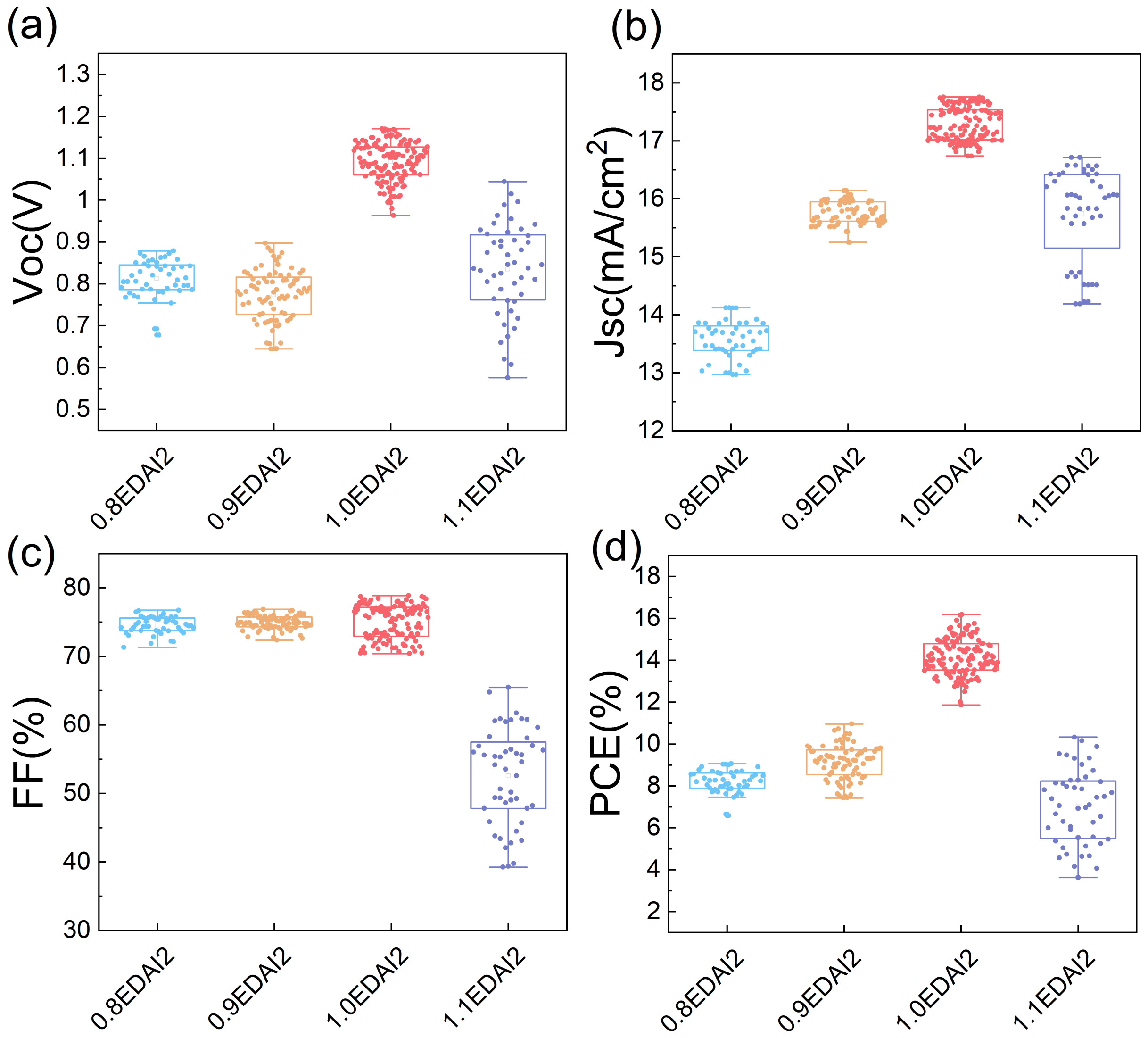

Fig. S11. Photovoltaic performance parameters: (a) V_{OC}, (b) J_{SC}, (c) FF, (d) PCE distribution of $CsPbI_3$ devices based on different $EDAI_2$ ratios ($Pb(OAc)_2$: CsI: $EDAI_2$=1.2:1:X, X=0.8, 0.9, 1.0, 1.1). A total of 320 devices were measured.

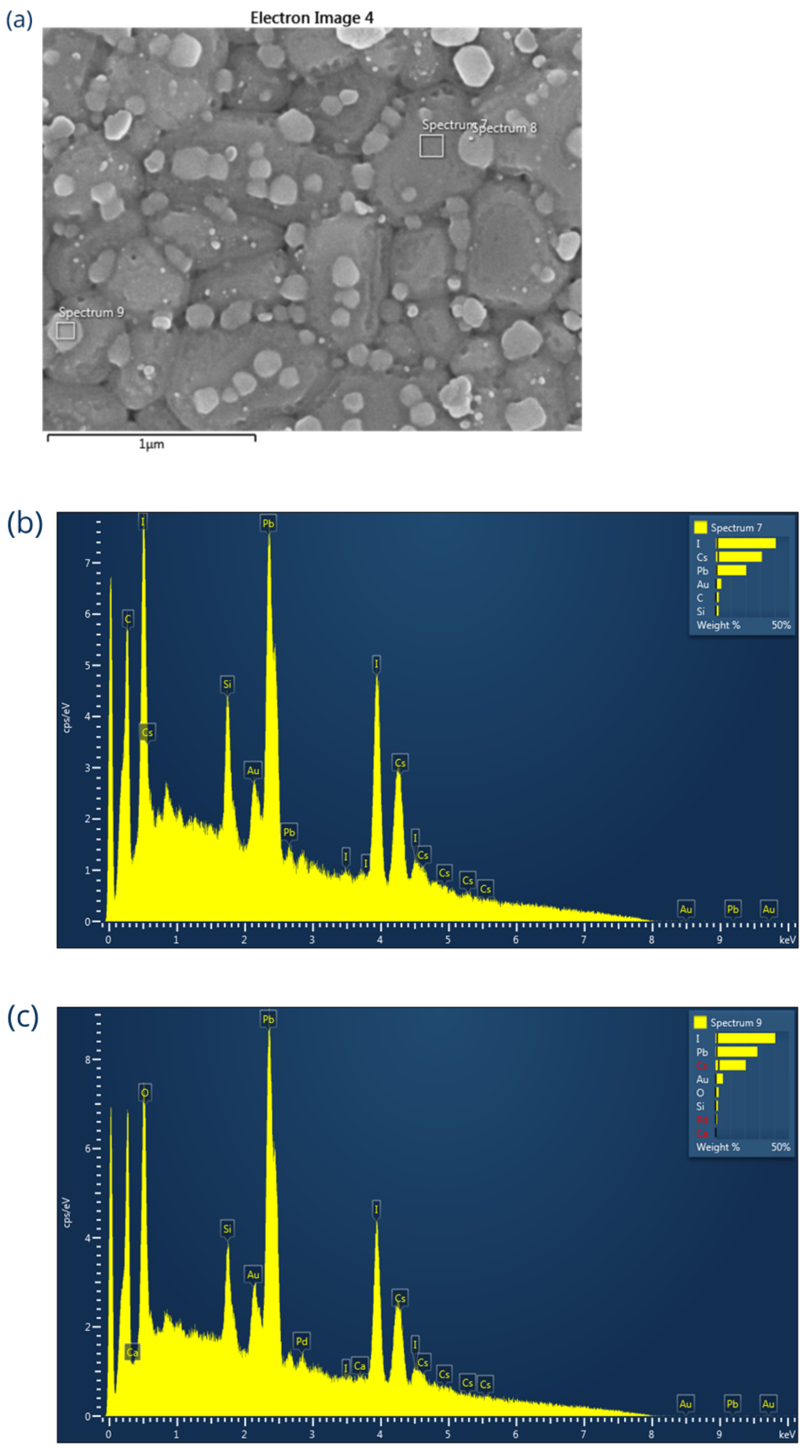

Fig S12. (a) Top-view SEM images and (b),(c) corresponding EDX element composition spectra in 1.2 samples on PTAA substrates.

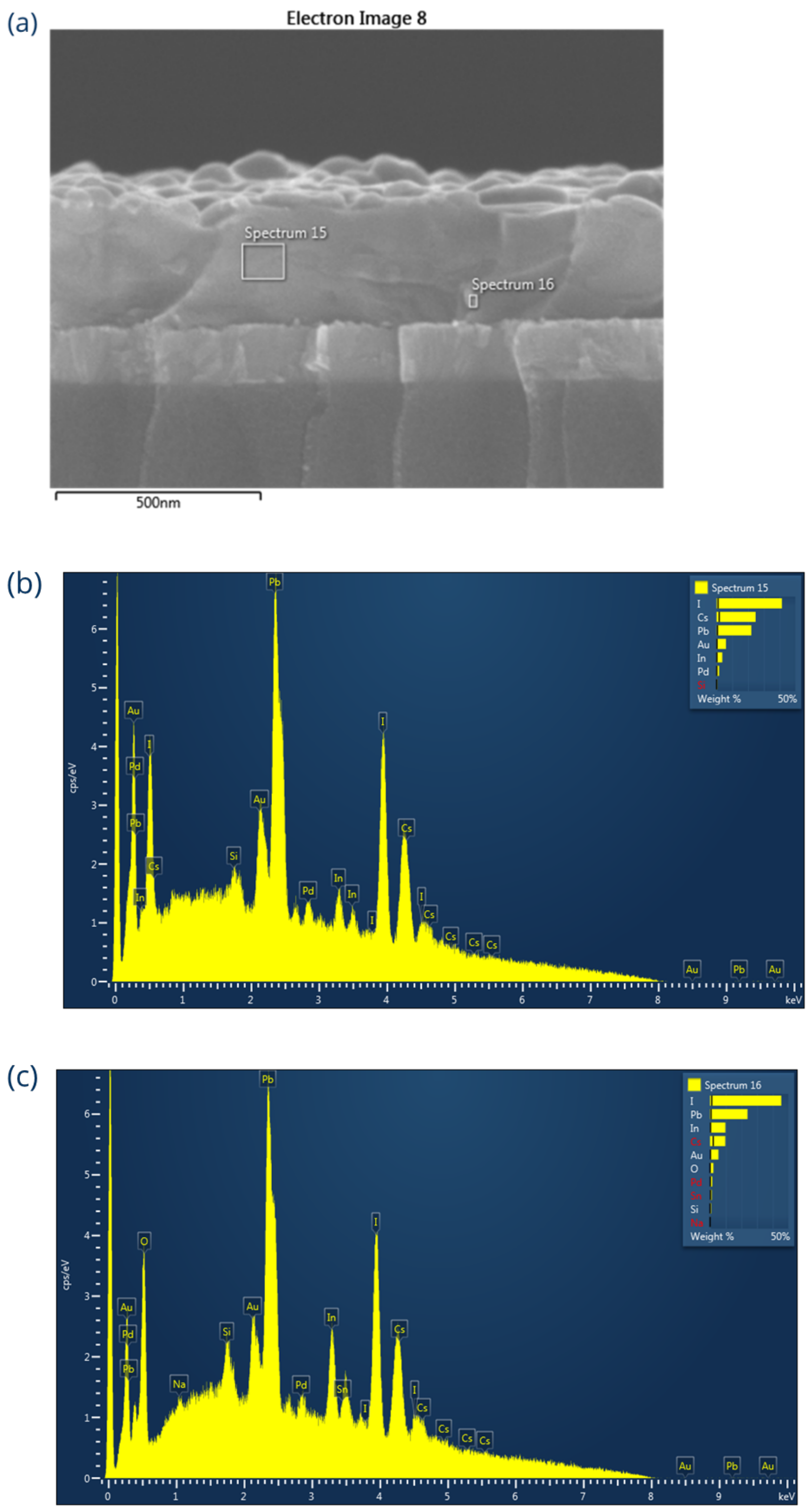

Fig S13.(a) Cross-sectional SEM images and (b)(c) corresponding EDX element composition spectra in 1.2 samples on PTAA substrates.

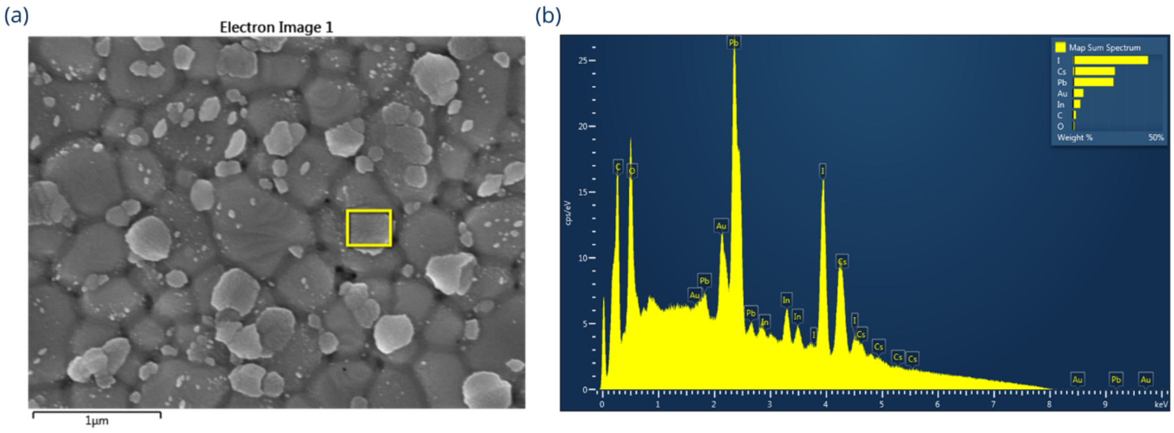

Fig S14. Top-view SEM images and corresponding EDX element composition spectra in 1.2 samples on MeO-2PACz substrates.

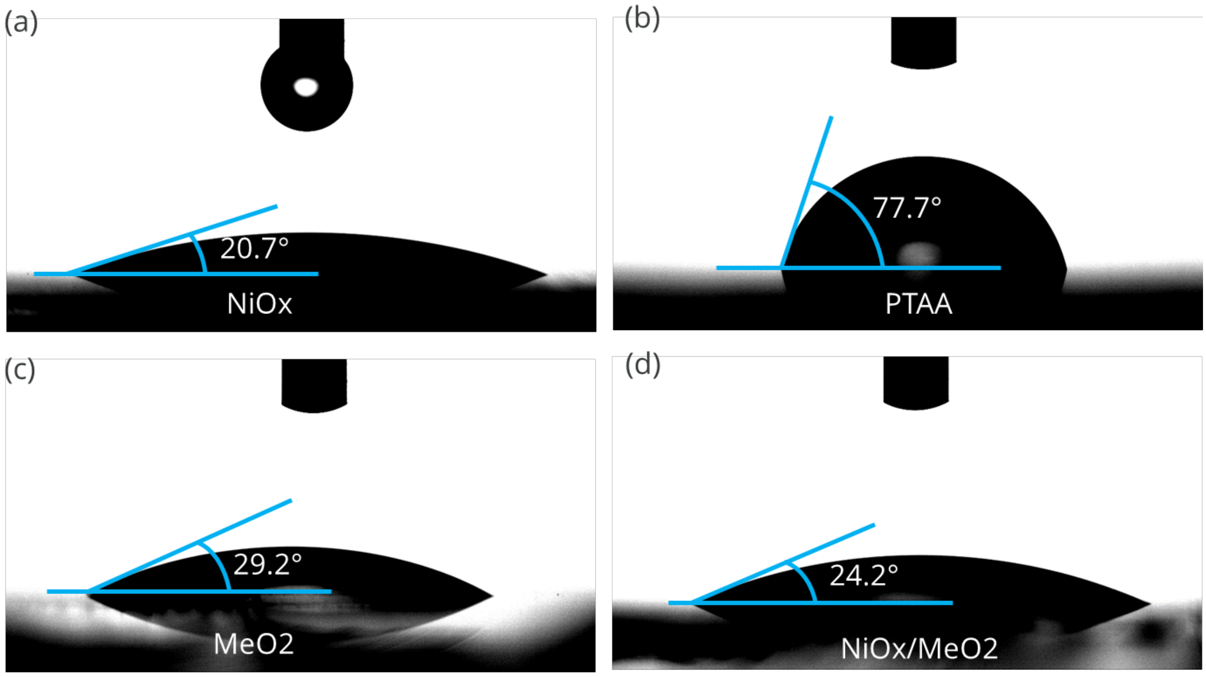

Fig. R15 Contact angle measurement of water on different HTLs: (a) NiOx, (b) PTAA, (c) MeO2 and (d) NiOx/MeO2 mixed hole transport layer.

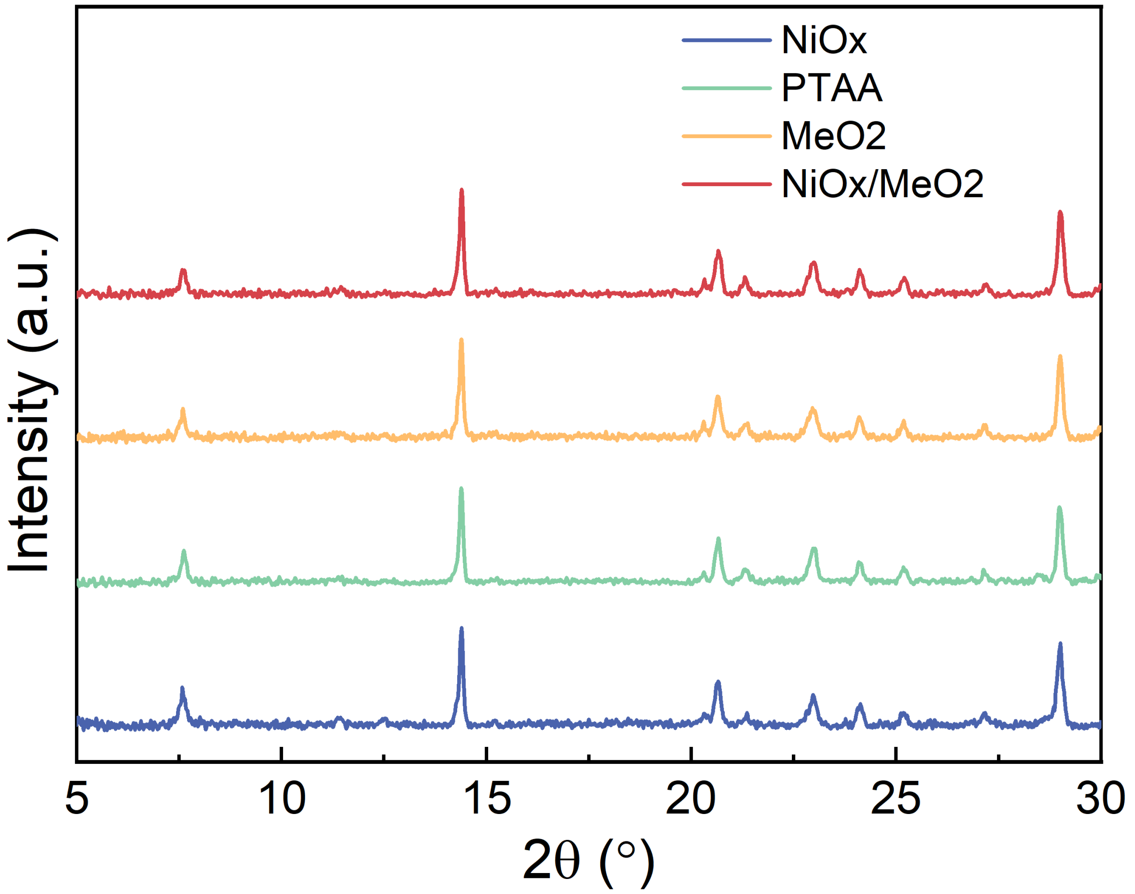

Fig. R16 XRD patterns of 1.2 $CsPbI_3$ on different hole transport layers.

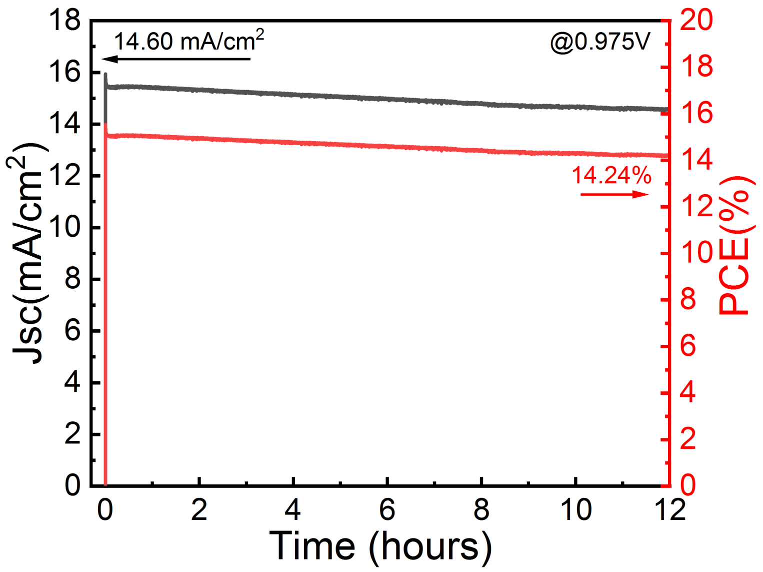

Fig S17. MPP tracking of 1.2 champion device on NiOx/MeO2 HTL.

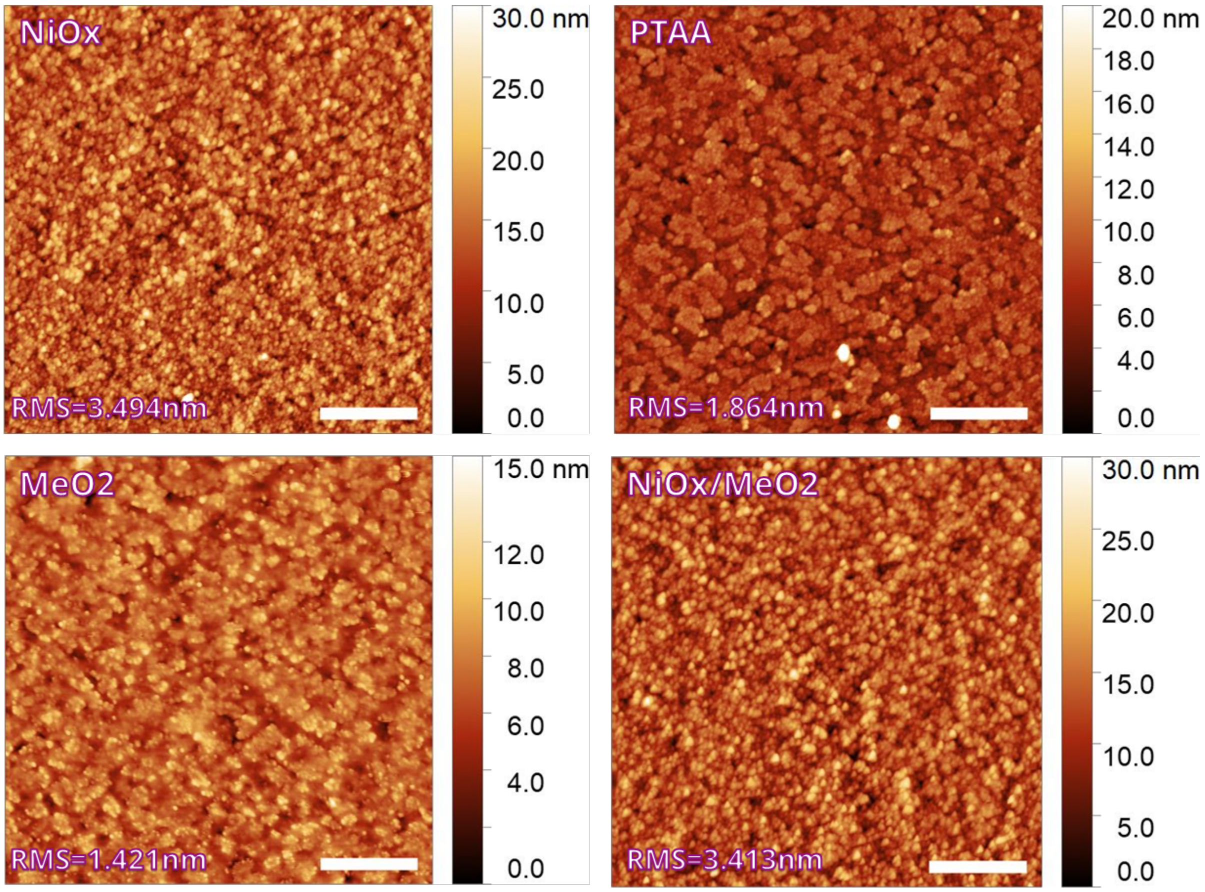

Fig S18. AFM images of different hole transport layer deposited on ITO substrates.

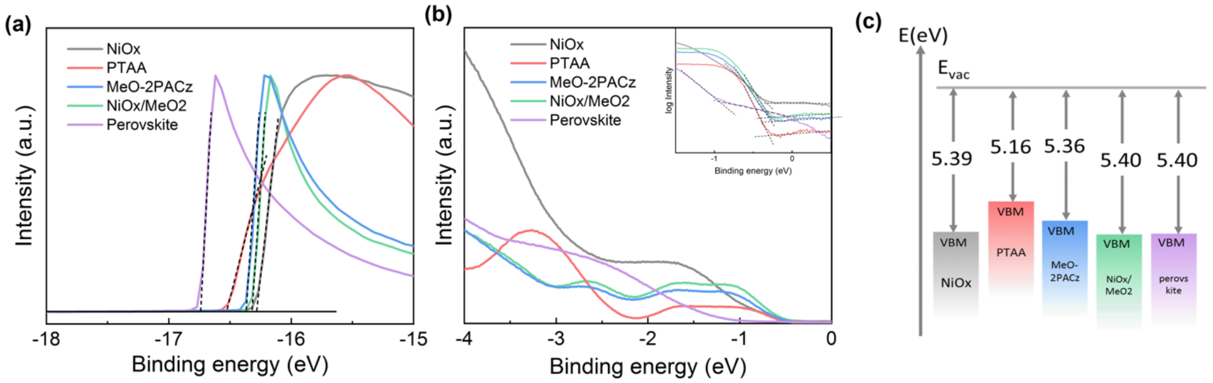

Fig S19. UPS spectra of 1.2 $CsPbI_3$ perovskite film, NiOx, PTAA, MeO-2PACz, NiOx/MeO2 deposited on ITO substrates. (a) SECO for determining the work function, (b) Binding energy range valence band spectra, (c) Energy level diagram for different HTLs and perovskite layer.

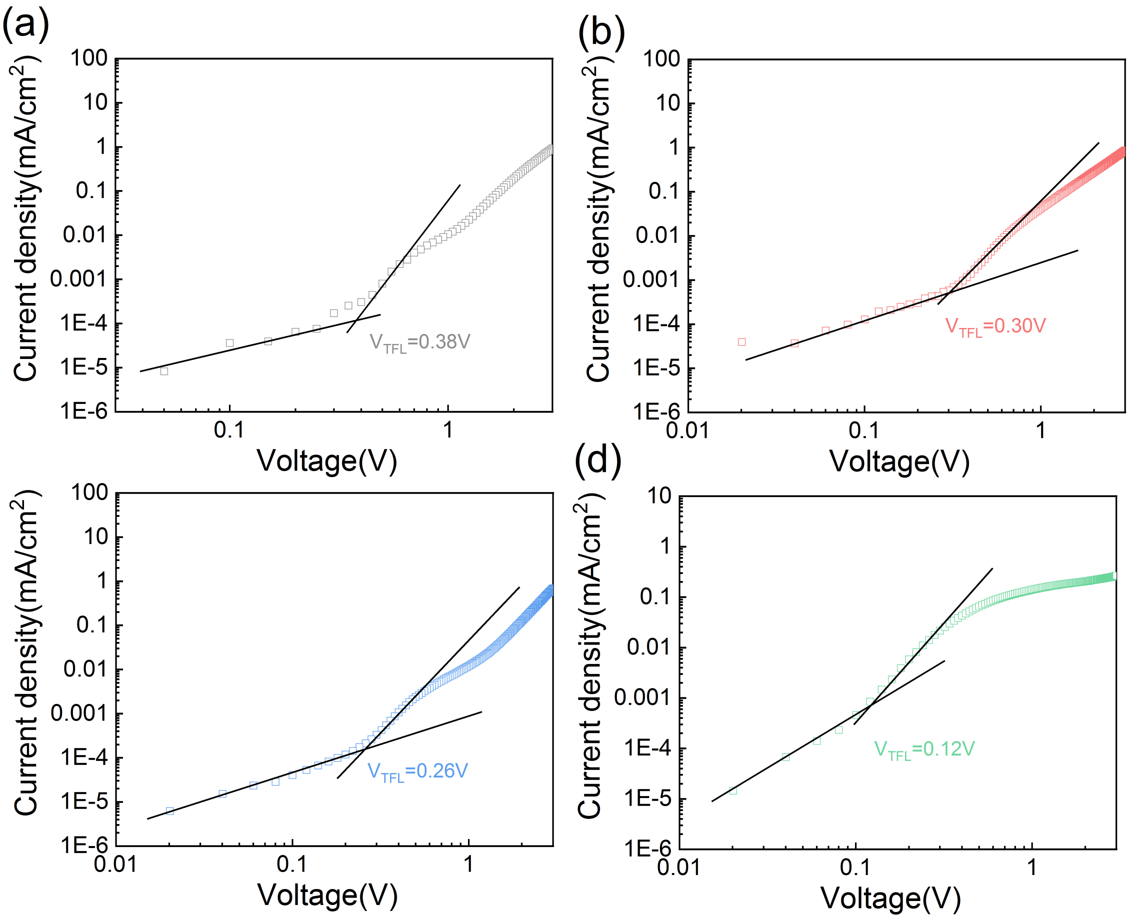

Fig S20. Dark I-V curves of perovskite devices on different hole transport layers: (a) NiOx, (b) PTAA, (c) MeO2 and (d) NiOx/MeO2. The device configuration is ITO/HTLs/perovskite/Spiro-OMeTAD/Au.

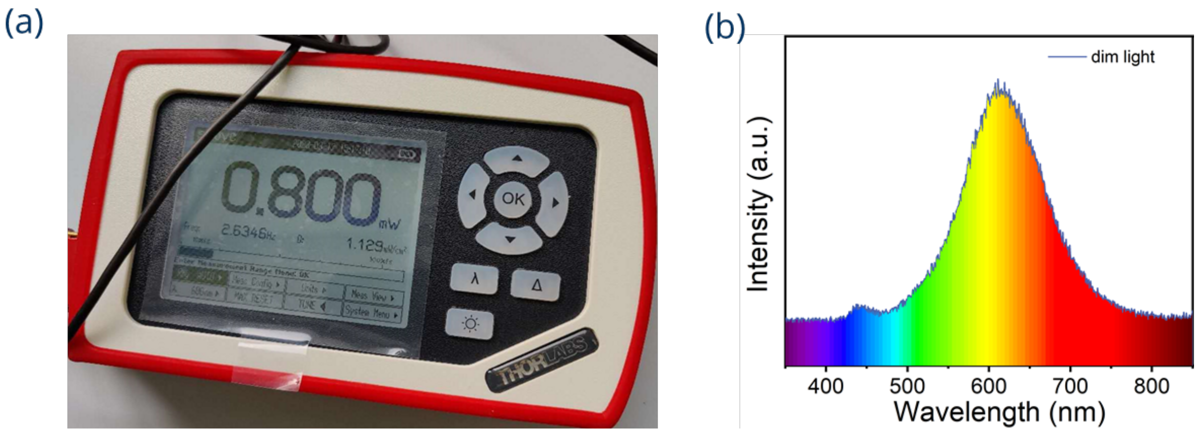

Fig. S21 Condition for shelf-storage measurement: (a) light intensity, (b) light spectrum.

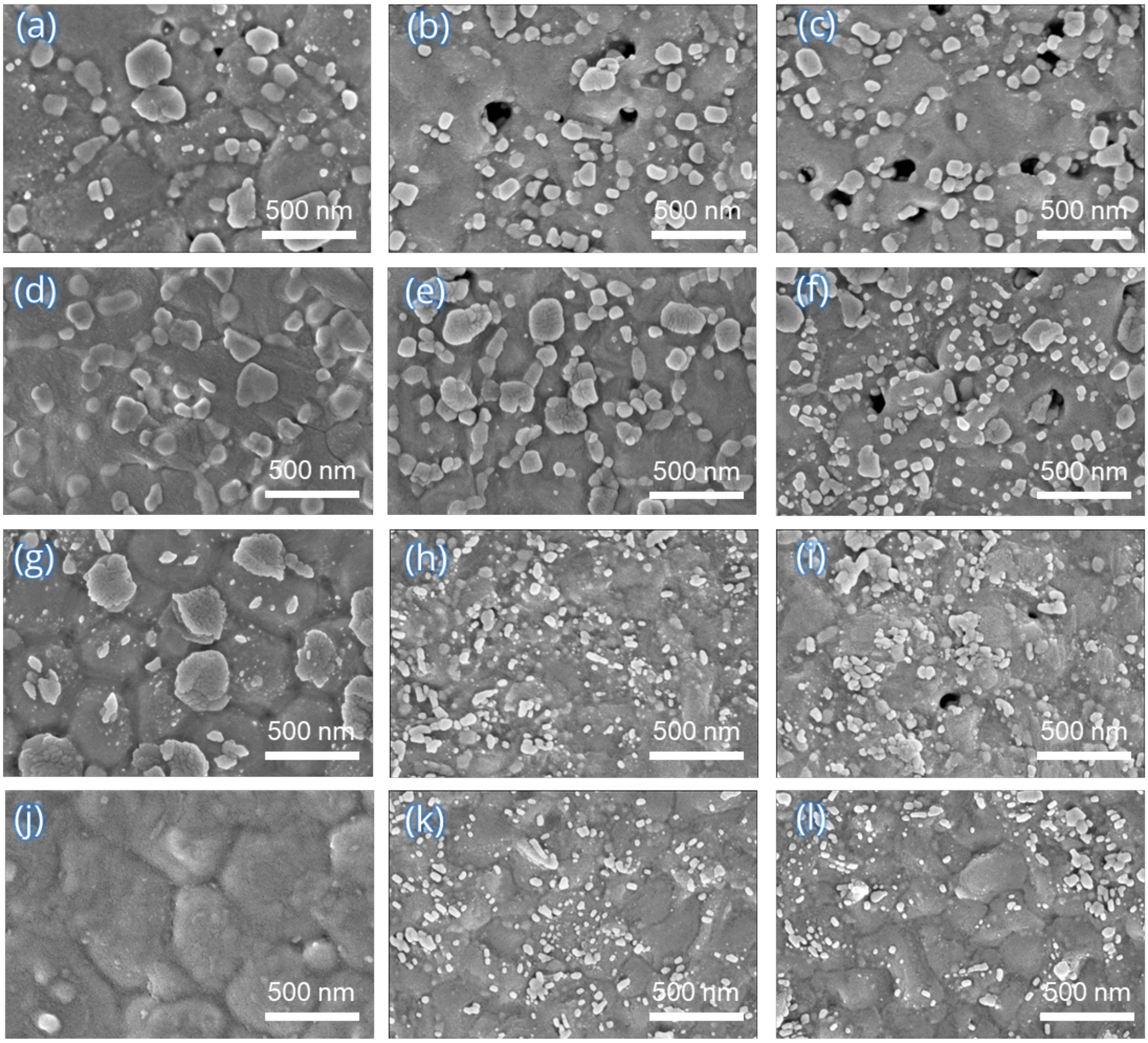

Fig. S22 SEM images of perovskite film on different HTLs under 1-sun illumination under nitrogen gas: NiOx with different time evolution (a) 0h, (b) 90h and (c) 200h; PTAA with different time evolution (d) 0h, (e) 90h and (f) 200h; MeO2 with different time evolution (g) 0h, (h) 90h and (i) 200h; NiOx/MeO2 with different time evolution (j) 0h, (k) 90h and (l) 200h.

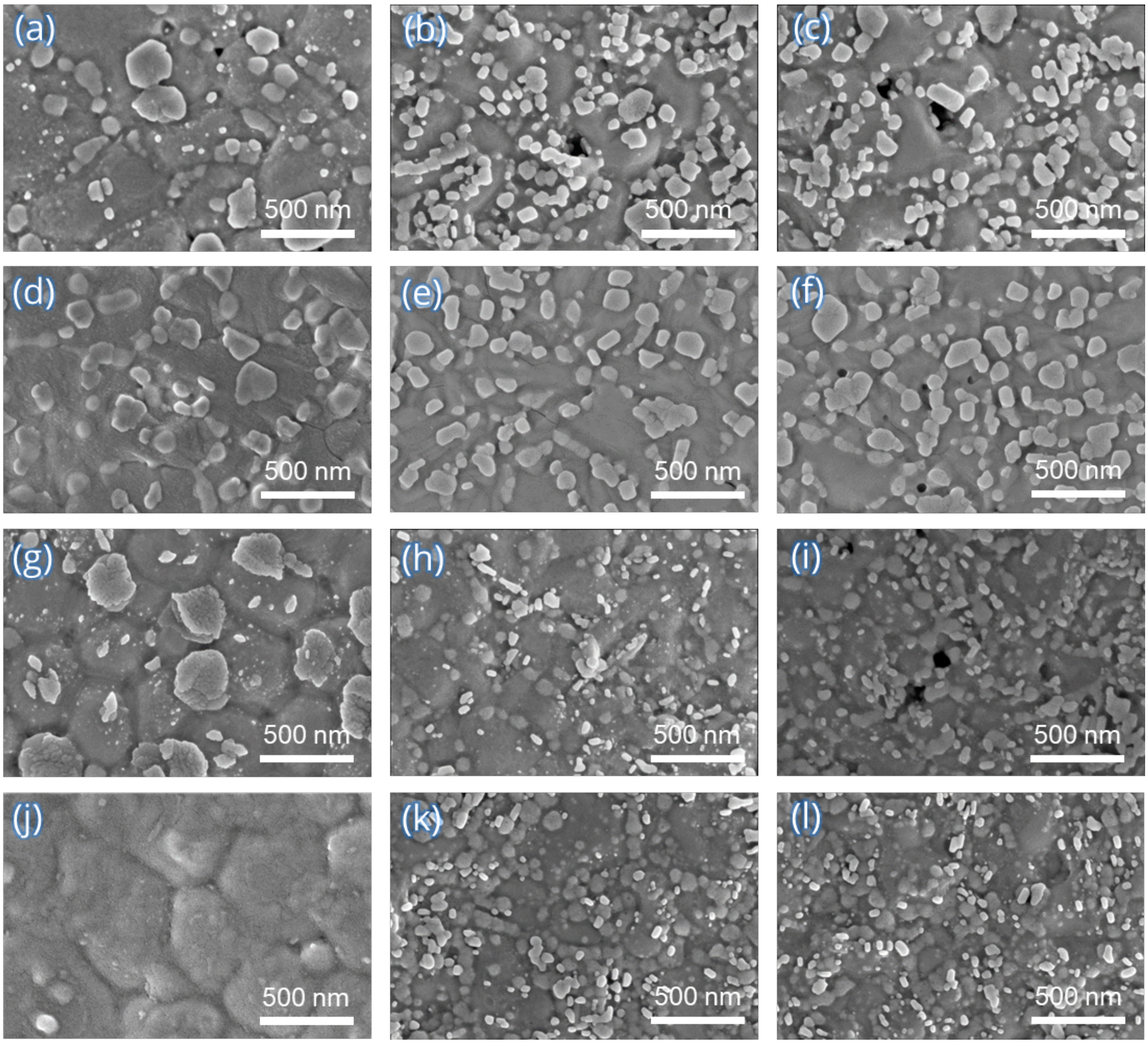

Fig. S23 SEM images of perovskite film on different HTLs under 1-sun illumination under nitrogen gas: NiOx with different time evolution (a) 0h, (b) 90h and (c) 200h; PTAA with different time evolution (d) 0h, (e) 90h and (f) 200h; MeO2 with different time evolution (g) 0h, (h) 90h and (i) 200h; NiOx/MeO2 with different time evolution (j) 0h, (k) 90h and (l) 200h.

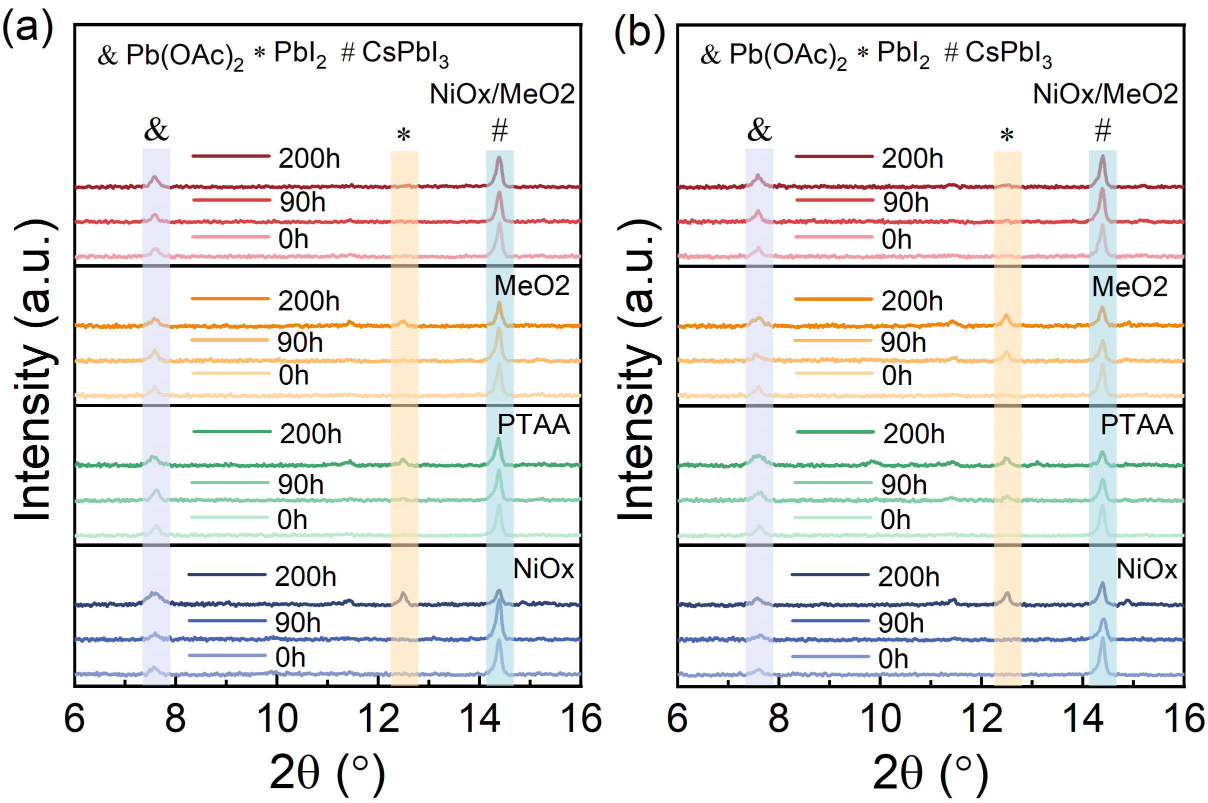

Fig. S24 XRD patterns of perovskite films on different substrates with time evolution under 1-sun illumination (a) and 80 °C heating (b).

Table S1 Element composition in 1.2Pb $CsPbI_3$ films varies with etching time

Etch Time (s)	Pb4f7 (%)	C1s (%)	O1s (%)	I3d5 (%)	Cs3d5 (%)
0	18.30	14.74	19.13	35.45	12.36
150	20.45	5.22	11.03	46.38	16.92
300	21.02	3.32	9.85	47.99	17.82
450	20.80	3.72	9.39	48.10	17.99
600	20.86	3.64	8.72	48.46	18.31

Table S2 Fitted carrier lifetime for different $Pb(OAc)_2$ excess

different Pb	A_1	τ_1/ns	A_2	τ_2/ns	A_3	τ_3/ns	τ_{ave}/ns
1.1	31286.82	0.84	8645.94	3.89	2837.27	10.54	5.21
1.15	12863.56	1.29	8321.98	4.92	2305.71	13.54	7.28
1.2	4603.23	2.08	8313.96	7.46	2224.01	18.26	10.91
1.25	14550.65	1.42	7725.36	5.01	1835.07	14.35	7.01
1.3	14393.03	1.15	8304.58	4.76	2510.24	12.32	6.76

Table S3 Photovoltaic performance parameters of champion cells with different $Pb(OAc)_2$ ratios (1.1, 1.15, 1.2, 1.25, 1.3)

Different Pb ratio	Voc [V]	Jsc [mA/cm^2]	FF [%]	PCE [%]
1.1-Forward	0.88	16.25	38.24	5.45
1.1-Reverse	0.84	16.25	55.32	7.57
1.15-Forward	1.07	16.83	66.15	11.93
1.15-Reverse	1.10	16.83	73.23	13.53
1.2-Forward	1.18	17.15	71.50	14.50
1.2-Reverse	1.20	17.15	76.60	15.81
1.25-Forward	1.00	16.64	68.71	11.42
1.25-Reverse	1.04	16.64	73.61	12.77
1.3-Forward	0.93	17.44	62.9	10.22
1.3-Reverse	0.97	17.44	66.04	11.12

Table S4 UPS spectra of different HTLs and 1.2Pb $CsPbI_3$ film

	WF (eV)	HOMO (eV)	IP (eV)
NiOx	4.93	0.46	5.39
PTAA	4.69	0.47	5.16
MeO2	4.85	0.51	5.36
NiOx/MeO2	4.90	0.50	5.40
Perovskite	4.46	0.94	5.40
